# Imaging heart development using high-resolution episcopic microscopy

**DOI:** 10.1016/j.gde.2011.07.004

**Published:** 2011-10

**Authors:** Timothy J Mohun, Wolfgang J Weninger

**Affiliations:** 1 MRC National Institute for Medical Research, London, UK; 2 Centre for Anatomy and Cell Biology, Medical University of Vienna, Austria

## Abstract

Development of the heart in vertebrate embryos is a complex process in which the organ is continually remodelled as chambers are formed, valves sculpted and connections established to the developing vascular system. Investigating the genetic programmes driving these changes and the environmental factors that may influence them is critical for our understanding of congenital heart disease. A recurrent challenge in this work is how to integrate studies as diverse as those of cardiac gene function and regulation with an appreciation of the localised interactions between cardiac tissues not to mention the manner in which both may be affected by cardiac function itself. Meeting this challenge requires an accurate way to analyse the changes in 3D morphology of the developing heart, which can be swift or protracted and both dramatic or subtle in consequence. Here we review the use of high-resolution episcopic microscopy as a simple and effective means to examine organ structure and one that allows modern computing methods pioneered by clinical imaging to be applied to the embryonic heart.

## Introduction

Human development, like that of other mammals, is critically dependent on the formation and function of the embryonic heart. Forming between 3 and 8 weeks of gestation, the heart supports subsequent growth of the foetus and it is perhaps not surprising that disruption of either heart development or function are believed to account for up to 10% of all miscarriages. Indeed, even amongst live births, anomalies of the heart are still detected in approximately 1% of babies and their management constitutes a significant medical burden. Heart development itself is an exquisitely complex process involving the transformation of a simple, tubular peristaltic pump into a mature, multi-chambered organ, capable of supporting separate systemic and pulmonary circulation upon birth. Understanding the complex interplay of growth, differentiation and tissue interactions and their underlying genetic programmes that drive formation of this organ is an enormous challenge for developmental biologists, but is essential if we are to unravel the environmental and genetic influences that result in congenital heart disease.

Animal models provide the opportunity both to examine normal heart development in a range of vertebrate embryos and to test the effect of experimental perturbation on heart morphogenesis or function. Structurally more similar to the human heart than that of avian or amphibian species, the mouse heart is most commonly used for studying cardiogenesis. Indeed, the past decade has witnessed a dramatic increase in our understanding of mouse heart development, driven primarily by the use of genetic manipulation. Not only has this facilitated study of the role played by individual genes in heart formation (revealing profound similarities in gene function between human and mouse counterparts), it has also provided the means to reliably distinguish the contribution of distinct cell lineages to the developing heart. As a result, the limiting factor is perhaps no longer the difficulty in establishing methods to perturb heart development; rather it is the challenge of integrating the burgeoning data from diverse studies of gene expression, cell lineage, proliferation and tissue architecture. Because of the complexity of its structure, the most informative way to examine such data has been to use the morphology of the developing heart as a three-dimensional scaffold on which other types of data can be superimposed. The models resulting from such synthesis have revealed many novel insights into heart morphogenesis and, by extrapolation to humans, have shed light on the likely origins of several cardiac malformations.

## Modelling heart development

Generating accurate 3D models of complex structures such as the embryonic heart is an age-old problem, initially addressed over a century ago using camera lucida techniques with microtome sections as the basis for wax models. Despite the many advances in imaging technologies including 3D imaging modalities that have transformed medical diagnosis, adapting these to analyse in the millimetre range necessary for embryos has proved challenging. As yet, neither magnetic resonance imaging nor the various tomographic methods (such as OPT and CT) can provide the resolution required to accurately model the changing morphology of the mouse heart over the course of embryonic development. The modern counterpart to the plate modelling of such nineteenth century pioneers as Born, His and Ziegler [1–3] remains remarkably similar: computer-based 3D rendering using realigned images of histological tissue sections. Paradoxically, although images of histological sections are unmatched in the extraordinary detail of tissue and cellular architecture they can reveal, much of this is lost from the 3D models produced by realigning sequential section images. This is a consequence of the variable and unpredictable distortions produced by tissue sectioning and staining and attempts to overcome this through choice of embedding medium, the inclusion of fiduciary markers or by computation have had only limited success [4–10,11^•^,12–17].

**Figure 1:**
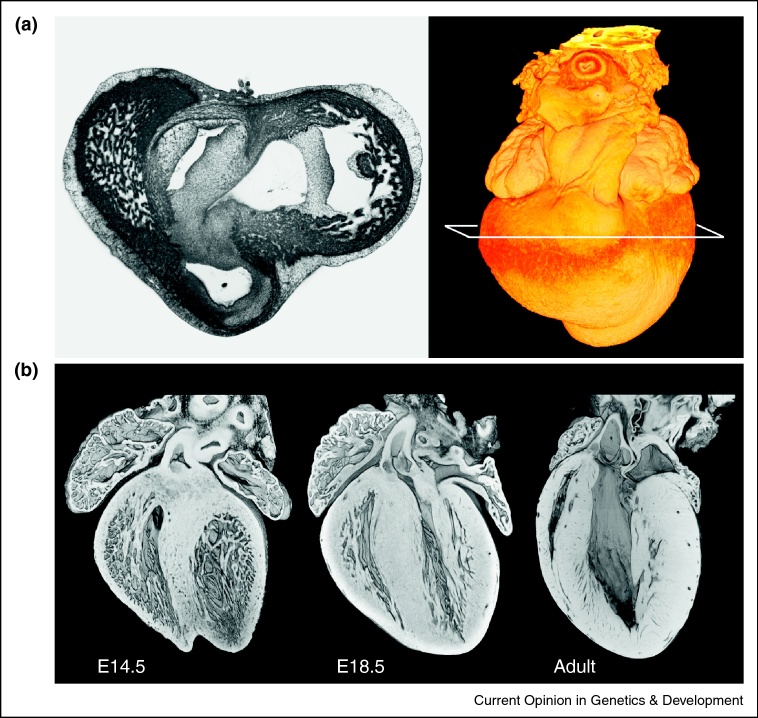
**(a)** An HREM image taken from an isolated chick embryo heart (HH stage 32) at the level of the developing atrioventricular junction, showing the range of grey levels associated with different tissue types. **(b)** 3D models of mouse embryo hearts isolated at E14.5 (when chamber septation is just completed) and E18.5 (shortly before birth) with that of the adult mouse. Models (not to scale) are eroded along a transverse plane from aortic valve to ventricular apex. This graphically illustrates the change in ventricular wall thickness and the mesh of spongy trabeculation that accompanies heart development.

Episcopic 3D imaging methods provide a solution to this problem, replacing individual section images with images of the embedded tissue block face [18^•^,19^•^,20–24]. High-resolution episcopic microscopy (HREM) has proved the most effective of these, using the simple expedient of fluorescent dyes in the plastic embedding medium to obtain very detailed greyscale images from a wide range of biological tissues and optical magnifications [25^••^]. For this reason it is particularly well suited to provide accurate data sets with which to explore the changing morphology of the developing heart (Figure 1a). Automation of a relatively rapid image capture cycle and the ability to choose inter-image distances as little as 1 μm with HREM equipment have several important benefits.

## The importance of numbers

Firstly, it is practical to analyse large numbers of samples. This is particularly helpful for analysing subtle or rapid developmental changes that make analysis of cardiac morphogenesis so challenging. Examples range from the developments in early cardiac chamber wall morphology resulting from the formation of trabeculae, transformation of the single outflow tract of the mid-gestation heart into distinct arterial trunks with remodelling of the associated aortic arch vessels [26], to changes in the mature embryo heart as it adapts to the change from uterine to terrestrial function at birth (Figure 1b). Similarly, analysis of numbers that would be unfeasible by conventional histology allows phenotypes that show variable or low penetrance to be investigated. It has, for example, been possible using HREM to investigate the precise range and type of cardiac malformations occurring in embryos of a trans-chromosomic mouse which incorporates the majority of human chromosome 21 as well as the normal diploid mouse genome. As a mouse model for studying human Down syndrome (DS), studies of this line are potentially compromised by low penetrance of the phenotype which may result from both tissue variability and mosaic retention of the human chromosome. Nevertheless, through studying sufficient numbers by HREM it has been possible to identify most of the same cardiac malformations seen in DS individuals, including the hallmark atrioventricular septal defect, albeit at relatively low prevalence [27^•^]. The same study used the high throughput possible with HREM to identify a significant difference in frequency of malformation between different mouse strain backgrounds.

Anecdotally, the contributory effect of strain background on phenotype is well known amongst researchers and has been noted in many studies, including those characterising cardiac phenotypes. Although it is both costly and difficult to characterise systematically, this may prove important for developing the accurate experimental models of human cardiac malformation or disease. Indeed, whilst differences between strains are known to affect animal husbandry, whether they have significant impact on aspects of normal development remains largely unexplored. Our own studies using HREM indicate that background strain and the degree of outbreeding can affect not only subtle effects on the relative timing of developmental changes during embryogenesis, but can also have profound qualitative and quantitative effects on aspects of cardiac morphology such as patterning or position of the coronary arteries and dimensions of the pharyngeal arch arteries [28].

## Measuring heart development

The detail provided by HREM images combined with the ability to manipulate entire data sets in 3D not only enables cardiac and vascular morphology to be visualised. It also allows accurate measurement of individual structures. To date, most analysis of heart development in the mouse has focussed on qualitative comparisons of normal and mutant hearts, usually using selected 2D histological sections. Quantitative measurements from such data are of course possible using techniques of unbiased stereology, but only if appropriately extensive and comparable section series are available. HREM analysis overcomes this problem by providing comprehensive data sets for each heart that can be digitally re-sectioned in the desired plane. Furthermore, direct volumetric measurement of individual structures is facile using modern 3D visualisation software packages (such as Amira [www.amiravis.com] and Osirix [www.osirix-viewer.com]) and is only limited by the task of selecting the desired region of interest. This opens the possibility of a more systematic and quantitative analysis of the changes in heart structure and composition during embryonic development. Not only would this resolve the extent of variation that may be inherent between individual embryos and the different mouse strains used in biomedical research, it may also provide an objective baseline for identifying developmental abnormalities that may be difficult to assess by qualitative criteria alone. For example, the normal range of variation in ventricular trabeculation is currently unknown. Grossly abnormal patterns have been identified in a few mutant mouse lines, which show embryonic lethality, but it is effectively impossible to identify milder phenotypes that might be helpful in analysing for example whether developmental aberrations underlie non-compaction disease.

**Figure 2:**
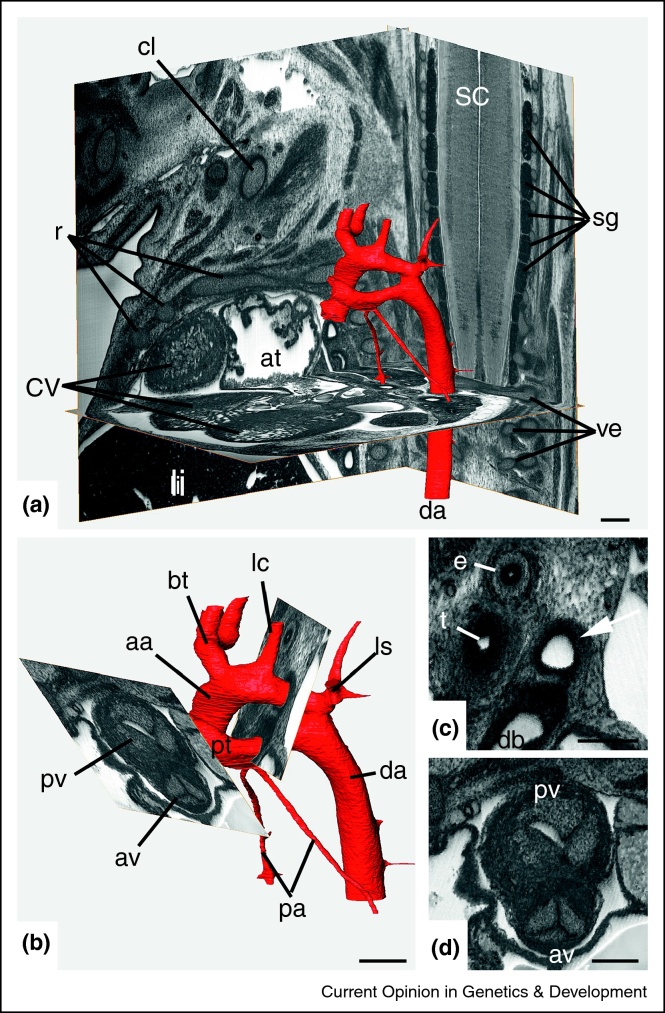
Measuring the diameters of the great intrathoracal arteries of a 14.5 dpc mouse foetus. **(a)** Great intrathoracic arteries (red) *in situ*. Surface rendered 3D models of the arteries are displayed together with the original HREM section plane and two re-section planes cutting perpendicular to each other and perpendicular to the original section plane through the HREM volume data. **(b)** 3D surface model and two oblique re-section planes. Note that the virtual planes were orientated to cut perpendicular to the longitudinal axis of the respective blood vessel segment through the original volume data. **(c)** and **(d)** Virtual section planes shown in (b). aa = ascending aorta, bt = brachiocephalic trunk, lc = left common carotid artery, ls = left subclavian artery, da = descending aorta, pt = pulmonary trunk, pa = pulmonary artery, db = ductus arteriosus (Botalli), av = aortic valve, pv = pulmonary valve, at = atrium, cv = cardiac ventricle, li = liver, r = rib, cl = clavicle, ve = forming vertebrae sc = spinal chord, sg = signal ganglion, t = trachea, e = oesophagus. Scale bar = 200 μm.

Similarly, HREM analysis has facilitated quantitative assessment of stenosis or dilation of the great intrathoracic arteries. Coarctation of the aorta or stenosis of the pharyngeal arch arteries and their derivatives often are associated with complex, intra-cardiac and extra-cardiac defects [e.g.] [29,30–33] which can result in prenatal or perinatal lethality. Accurate detection of stenosis in embryonic and foetal blood vessels requires histological sections cut precisely perpendicular to the longitudinal axis of the artery being measured. Technically challenging with adult mice, this conventional approach is impossible with mouse embryos. Its digital equivalent is however straightforward with image volume data — and only HREM data currently provides spatial resolution adequate to yield meaningful measurements [34,35] (Figure 2).

## Visualising cardiac gene expression

**Figure 3:**
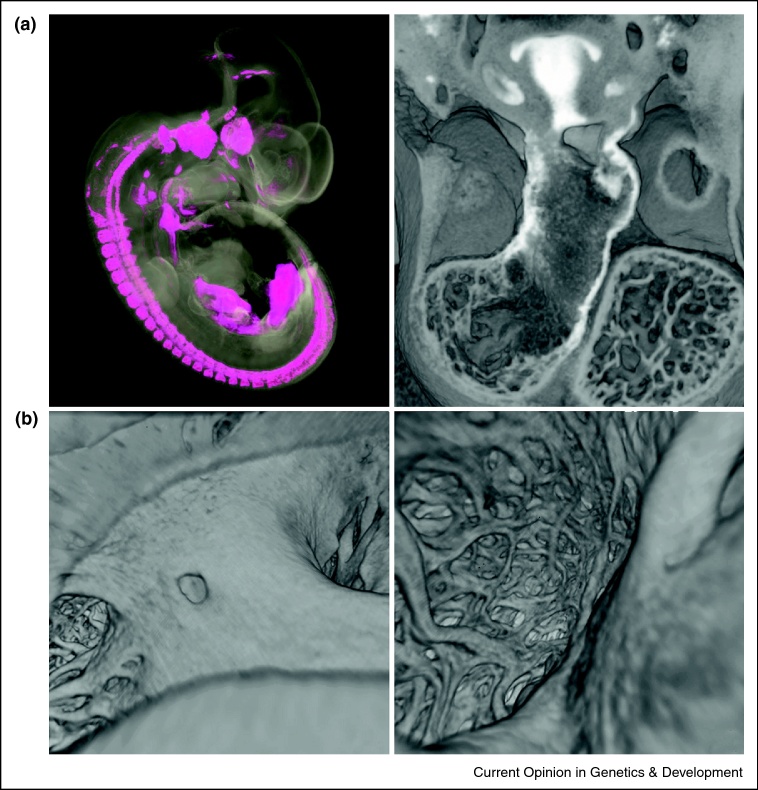
**(a)** 3D models of β-galactosidase expression in an E11.5 Islet1-lacZ embryo, captured by dual wavelength HREM and pseudo-coloured magenta in the whole embryo (left) or white in the heart (right). The latter is eroded in the transverse plane to show expression in the central pharyngeal region (including the dorsal roof of the aortic sac) as well as both proximal and distal walls of the outflow tract. **(b)** Images obtained by ‘virtual endoscopy’ using HREM data from an E18.5 mouse embryo heart, revealing remarkable details of heart structure. The left panel shows a view of the right atrium from the entrance of the right superior caval vein. Note the smooth ventral floor of the right atrium surrounding the coronary sinus, flanked on one side by the trabeculae of the right atrial appendage and edge of the tricuspid valve on the other. The right panel shows a view of the trabecular lattice within the right atrium, viewed through the tricuspid valve.

3D modelling of gene expression patterns has had an important impact on our understanding of heart morphogenesis by revealing the contributions of different cell lineages either directly (using CRE-mediated recombination to activate reporter genes) or indirectly (using endogenous gene expression patterns as a surrogate for lineage marking). As more marker genes for cardiac cells and tissues are identified, such studies will increasingly allow all aspects of cardiac development to be reassessed. Gene expression studies have almost exclusively relied on staining individual sections, since this has yielded the most sensitive results and allowed investigation of several gene patterns simultaneously. However, as with studies of morphology, reconstruction of the expression data into 3D models inevitably results in significant loss of resolution, in part from the limited frequency of sections but also from the constraints imposed by poor section registration. The finding that HREM can be adapted to detect localised patterns of gene expression revealed by colourimetric stains is therefore potentially important [25^••^]. Of course, the gains obtained from episcopic imaging may be offset by the loss of signal sensitivity resulting from wholemount rather than section staining procedures. This is undoubtedly the case for later stages of heart development in the mouse where penetration of staining reagents into dense cardiac tissue can be problematic. However, for stages of development up to E11.5–12.5, covering much of the period during which the heart is formed, reasonable staining appears possible and the resulting data can be combined with morphology to produce highly detailed 3D models (Figure 3a).

## Future prospects

With the rapid increase in availability of genetically altered mouse lines (e.g. from systematic gene knockout programmes such as EUCOMM and KOMP), a consistent and sensitive method for identifying cardiac malformations in mouse embryos is essential [36^•^]. In the absence of adequate, non-destructive 3D imaging methods, HREM provides a simple way to achieve this. The 3D data sets of morphology and gene expression it provides can be explored with modern imaging software, yielding powerful and novel ways to examine cardiac morphogenesis (Figure 3b).

## References and recommended reading

Papers of particular interest, published within the period of review, have been highlighted as:

• of special interest
•• of outstanding interest
